# Challenges in a Hybrid Fabrication Process to Generate Metallic Polarization Elements with Sub-Wavelength Dimensions

**DOI:** 10.3390/ma13225279

**Published:** 2020-11-22

**Authors:** Stefan Belle, Babette Goetzendorfer, Ralf Hellmann

**Affiliations:** Applied Laser and Photonics Group, Aschaffenburg University of Applied Sciences, Wuerzburger Str. 45, 63743 Aschaffenburg, Germany; babette.goetzendorfer@th-ab.de (B.G.); ralf.hellmann@th-ab.de (R.H.)

**Keywords:** hybrid fabrication process, three-dimensional direct laser writing, hollow waveguide array, metallic sub-wavelength structures, quarter- and half-wave plate

## Abstract

We report on the challenges in a hybrid sub-micrometer fabrication process while using three dimensional femtosecond direct laser writing and electroplating. With this hybrid subtractive and additive fabrication process, it is possible to generate metallic polarization elements with sub-wavelength dimensions of less than 400 nm in the cladding area. We show approaches for improving the adhesion of freestanding photoresist pillars as well as of the metallic cladding area, and we also demonstrate the avoidance of an inhibition layer and sticking of the freestanding pillars. Three-dimensional direct laser writing in a positive tone photoresist is used as a subtractive process to fabricate free-standing non-metallic photoresist pillars with an area of about 850 nm × 1400 nm, a height of 3000 nm, and a distance between the pillars of less than 400 nm. In a subsequent additive fabrication process, these channels are filled with gold by electrochemical deposition up to a final height of 2200 nm. Finally, the polarization elements are characterized by measuring the degree of polarization in order to show their behavior as quarter- and half-wave plates.

## 1. Introduction

Laser material processing technologies comprise innumerable subtractive and additive manufacturing approaches. Typical subtractive, material-removing, processes are, e.g., laser modification and removal [1,2], laser ablation [3,4], laser micro-drilling [5,6], laser cutting [7,8], and laser structuring [9,10]. Laser-based additive manufacturing encompasses, most commonly, stereolithography [11,12], selective laser melting [13,14,15], laser sintering [16,17], or laser metal deposition [18,19]. In addition to these, predominantly for macroscopic components employed technologies, additive manufacturing has been advanced to a technology that is capable of generating structures in the sub-micrometer range through the use of ultrashort pulse lasers. Multiphoton lithography or also known as three-dimensional direct laser writing (3D DLW) uses a tightly focused laser beam from an ultrashort pulse laser to excite a polymerization process in a photosensitive material by multiphoton absorption. The photosensitive material is usually a negative tone photoresist or a hybrid polymer. A three-dimensional movement of the sample and/or of the beam creates three-dimensional complex structures inside the photosensitive material by a pinpoint writing process [20,21,22,23,24].

**Figure 1:**
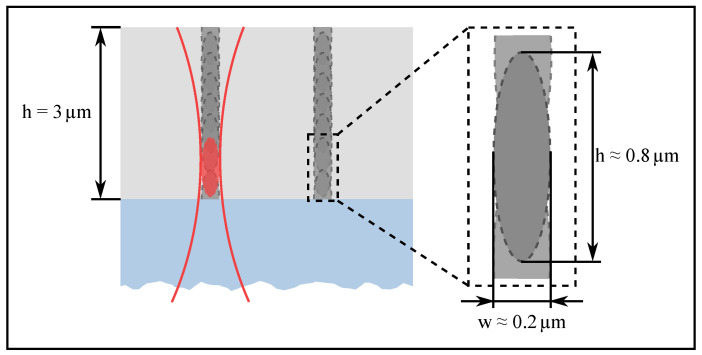
Schematic drawing of a three-dimensional direct laser writing (3D DLW) writing process. The blue area in the figure depicts the substrate, the light gray area the unexposed photoresist, the dark gray areas the exposed photoresist, the red lines approximate the envelope of the laser beam used for exposure, which is strongly focused by an objective with a high numerical aperture and the red area illustrates the focal volume (voxel) in which the polymerization occurs. The right side shows an enlarged illustration of a voxel with the approximate dimensions.

In this work, the advantages of three-dimensional direct laser writing are used, but the process itself is inverted. By using a positive tone photoresist instead of a negative tone photoresist, the exposed areas are removed after developing and the additive fabrication process becomes a subtractive fabrication process. The advantages of 3D DLW, such as the high lateral resolution or the exposure of a limited volume area, are transmittable to the subtractive process and they are necessary for a successful fabrication. Figure 1 shows a schematic drawing of the three-dimensional direct laser writing in a positive tone photoresist. On the left side of Figure 1, the shape and dimensions of the focal volume are shown. Having a width of about 200 nm and height of about 800 nm for the given objective with a numerical aperture of 1.4, the polymerized focal volume (voxel) is not large enough for an exposure of the entire height of the photoresist. Therefore, the photoresist must be exposed several times in different levels. By using a lens with a lower numerical aperture, a complete exposure of the photoresist can be achieved. However, this is accompanied by a reduction in the lateral resolution so that the fabrication of photoresist pillars with sub-micrometer dimensions is not possible and the transmission of the final hollow waveguide array would also be reduced. Despite the advantages mentioned, only a few examples of the combination 3D DLW with positive tone photoresist can be found in the literature [25,26]. The combination of the subtractive fabrication process, three-dimensional direct laser writing, with the additive fabrication process, electrochemical metal deposition, to a hybrid manufacturing process enables the fabrication of components with extraordinary functions [27,28].

Besides three-dimensional direct laser writing, several methods are conceivable for fabricating such a structure. Metamaterials for the visible wavelength range are fabricated mainly with electron beam lithography (EBL). EBL has a high lateral resolution of a few tens of nanometers, but it has disadvantages in terms of thick resist layers due to electron scattering and the writing speed is low [29,30]. For these reasons, EBL is therefore not suitable for the fabrication of the polarization elements presented in this work. Standard UV lithography is also not suitable for fabrication. The minimum line width that is achieved by contact exposure is approximately by a factor of two larger and accordingly reduces the transmission properties of the components [31]. Deep mask-based lithography is a suitable manufacturing process, the achievable resolution is correspondingly high due to the short wavelength of the used source, and, due to the parallel exposure of all structures, is significantly faster than 3D DLW. Disadvantages of this method, however, are the complex mask fabrication for the deep UV exposure and the limited access to a synchrotron radiation source [32]. We decided to use three-dimensional direct laser writing for fabrication due to the advantages of 3D DLW mentioned in the previous paragraph and the arguments listed here.

The aim of this paper is to show and discuss the challenges of this hybrid subtractive and additive fabrication process by demonstrating a polarization converter as an exemplifying macroscopic component with sub-micrometer function determining the spatial features. In particular, a quarter- and a half-waveplate are manufactured, both being based on the concept of hollow waveguides, as theoretically described by Helfert et al. and recently experimentally demonstrated by the authors [33,34,35].

## 2. Basic Theory of Hollow Waveguides

**Figure 2:**
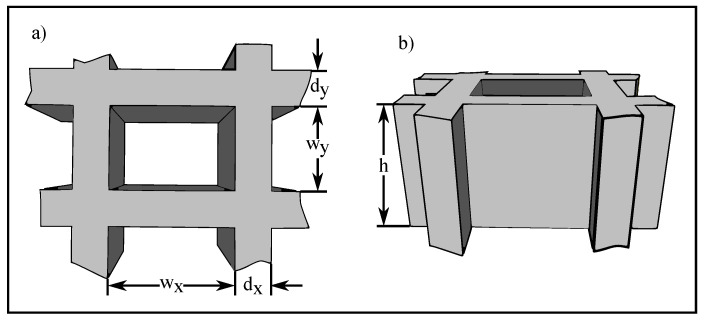
Schematic drawing of a hollow waveguide: (**a**) shows a top view and (**b**) shows a tilted side view of a hollow waveguide. The grey-colored structure are the surrounding gold sidewalls. The rectangular spacing in between is the air-filled hollow waveguide.

A hollow waveguide consists of a dielectric medium, in the simplest case ambient air, surrounded by a metallic wall, for example, gold. The lateral dimensions of a hollow waveguide are on a sub-wavelength scale. This dimensions makes it necessary to arrange the hollow waveguides in a field consisting of thousands in order to control the polarization state of a laser beam. Figure 2 illustrates one single waveguide.

Using a HWA as a polarization converter for different propagation constants of the vertical and horizontal modes is necessary. This is achieved by using waveguides with a rectangular cross-section. The effective refractive index, which is related to the propagation constant, of these modes can be calculated by [33]

(1) $$n_{eff,10}=\sqrt{1-\frac{\lambda_{0}^{2}}{4w_{x}^{2}}}$$

and

(2) $$n_{eff,01}=\sqrt{1-\frac{\lambda_{0}^{2}}{4w_{y}^{2}}}$$

with $n_{eff}$ as the effective refractive index of the horizontal and vertical modes, $\lambda_{0}$ as the free-space wavelength, and $w_{x,y}$ as the width of one single hollow waveguide. The phase delay between the two modes can be determined by

(3) $$\Delta \Phi =\frac{2\pi (n_{eff,10}-n_{eff,01})h}{\lambda_{0}}$$

where *h* is the height of the HWA. During the fabrication, the height and the width $w_{x}$ of the hollow waveguides is fixed at *h* = 2200 nm and $w_{x}$ = 1400 nm. To achieve a phase delay of 270${}^{\circ }$ (quarter-wave plate) for a free-space wavelength of 1550 nm, a width of $w_{y}$ = 814 nm is necessary and a width of $w_{y}$ = 884 nm for a phase delay of 180${}^{\circ }$ (half-wave plate).

## 3. Fabrication

**Figure 3:**
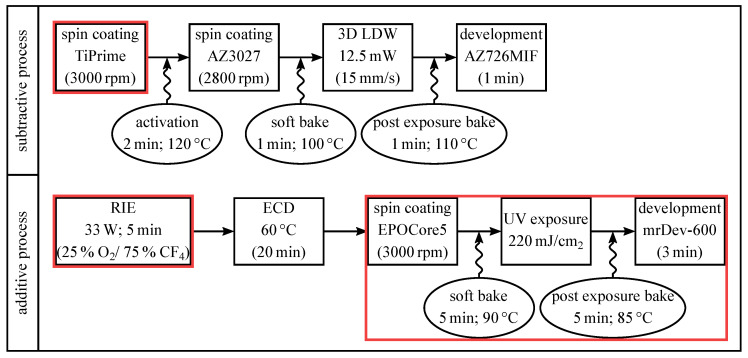
Process chain for the two sub-processes: Top, the fabrication of the negative structure using 3D LDW and a positive tone resist as a subtractive process. Bottom, electrochemical deposition (ECD) of gold as an additive process. The fabrication steps that are marked with a red rectangle are the most challenging process steps during fabrication. These steps are discussed in detail in Section 4, because of their particular importance in the hybrid fabrication process.

The hybrid fabrication process of the polarization element can be divided into two sub-processes. First, the subtractive process for fabricating a negative structure, the non-metallic freestanding photoresist pillars, of the final HWA, and, second, the electrochemical deposition of the metallic sidewalls as an additive process. Figure 3 depicts the complete process chain of the two sub-processes. Please note, the following description of the fabrication process in this chapter is based on the publication of two of the authors in [35] and enriched with further details.

For the fabrication of the negative structure, a clean indium tin oxide (ITO) coated glass substrate (Zeiss AG, Oberkochen, Germany) is baked at 140${}^{\circ }$ for 15 min. for the desorption of residual water molecules deposited on the surface. After this first baking step, an adhesion promotor TiPrime (MicroChemicals GmbH, Ulm, Germany) is spin coated onto the ITO coated glass substrate at 3000 rpm and it is activated in a subsequent, second baking step at 120${}^{\circ }$ for 2 min. Afterwards, the positive tone photoresist AZ3027 (MicroChemicals GmbH, Germany) is applied by spin coating at 2800 rpm. For mechanical and chemical stabilization of the spin coated layer, the sample is baked on a hotplate at 100${}^{\circ }$ in order to reduce the solvent content of the photoresist. Following, the photoresist is exposed with a 3D DLW-setup Photonic Professional GT (Nanoscribe GmbH, Karlsruhe, Germany). The 3D DLW setup provides 100 fs pulses at 780 nm with a repetition rate of 80 MHz. Here, the writing speed is 15 mm/s with an average laser power of 12.5 mW. A total of 36 × 36 individual fields with the dimensions of 100 μm × 100 μm for each field are exposed in order to obtain a macroscopic component. The writing time for one field with the writing speed of 15 mm/s is 25 s. Immediately after exposure, the resist film is baked at 110${}^{\circ }$ for 1 min and then developed in a ready to use developer AZ726MIF (MicroChemicals GmbH, Germany) for 1 min. The developed photoresist layer is thoroughly rinsed with deionized water [35].

**Figure 4:**
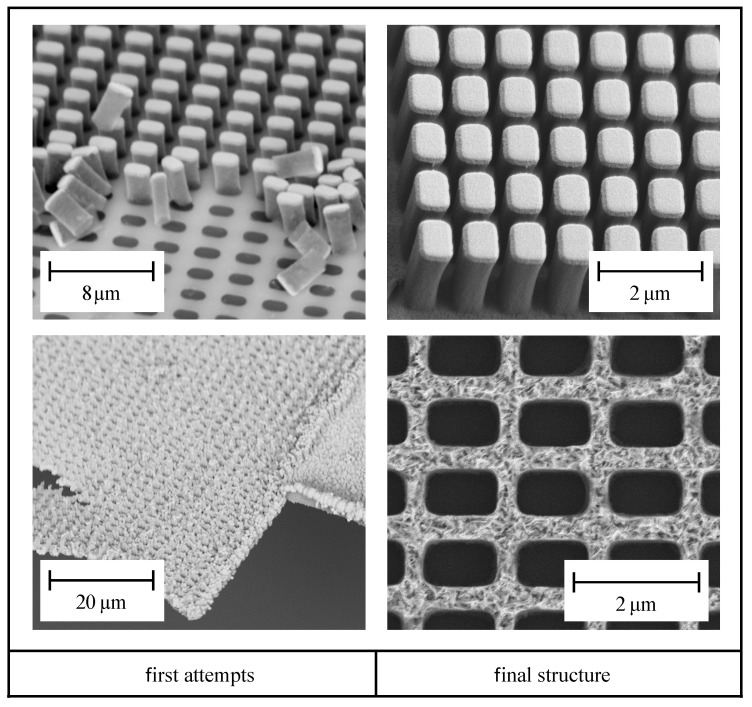
SEM images to compare the challenges from the first attempts (**left**) to the final fabrication (**right**). The top images show the freestanding pillars resulting from the subtractive process and the bottom images the final HWA structure.

After developing the resist film, a disturbing thin inhibition layer is formed on the ITO layer. This thin layer prevents contact between the ITO and the electrolyte and must, therefore, be removed. The additive sub-process begins with the removal of this inhibition layer with an anisotropic reactive ion etching (RIE) step. Anisotropic RIE is performed with a gas mixture of 25 % O_2_ and 75 % CF_4_, in a 40 kHz RIE setup Pico (Diener Electronic GmbH, Ebhausen, Germany) with a generator power of 30 W for 5 min. Further, the ITO coated glass substrate with the freestanding photoresist pillars is built into an electrochemical deposition (ECD) chamber and it is placed onto a heating plate at 60${}^{\circ }$. To achieve a smooth cladding surface and a good mechanical stability, the ECD is performed by alternating pulsed and constant current. The total time for electrochemical deposition is about 20 min., resulting in a gold height of 2200 nm. In order to enhance the adhesion of the gold layer on top of the ITO, an EpoCore5 negative tone photoresist (MicroResist GmbH, Berlin, Germany) is spin coated with a rotational speed of 3000 rpm over the entire structure and softbaked at 90${}^{\circ }$ for 5 min. Subsequently, the photoresist is exposed by UV-light of a mask aligner EVG620 (EVG GmbH, St. Florian am Inn, Austria) at a dose of 220 mJ/cm^2^. After exposure, a hardbake is done at 85${}^{\circ }$ for 5 min. and the sample is developed in a ready to use developer mr-Dev 600 (MicroResist GmbH, Germany) for 3 min. With this last developing step, a circular area of 2 mm in diameter is opened and within this circular area also the freestanding pillars are completely removed by the developer. The freestanding photoresist pillars and the final HWA are both shown in Figure 4, right column [35].

## 4. Fabrication Challenges

However, the subtractive and additive hybrid process for the fabricaton of metallic optical elements with sub-wavelength dimensions is hallmarked by some challenging process steps, which are marked by red rectangles in the process chain depicted in Figure 3 and that are discussed in detail in the following subsections. In order to illustrate these challenges and, thus, to clarify the importance of the marked process steps for a successful fabrication, Figure 4 contrasts the effects of sub-optimal process conditions (at the beginning of our study on the left) and optimized process settings (final structures on the right).

### 4.1. Weak Adhesion (Part I)

In order to improve the rather weak adhesion of the freestanding photoresist pillars (Figure 4, top (left)), several treatments of the ITO coated substrate were tested (namely, different combinations of RIE, heat treatment and coating with an adhesion promotor, respectively) and compared to completely untreated substrate properties. Worthwhile to mention, besides improving the adhesion of the photoresist, it is also important to not damage the conductive ITO layer.

**Figure 5:**
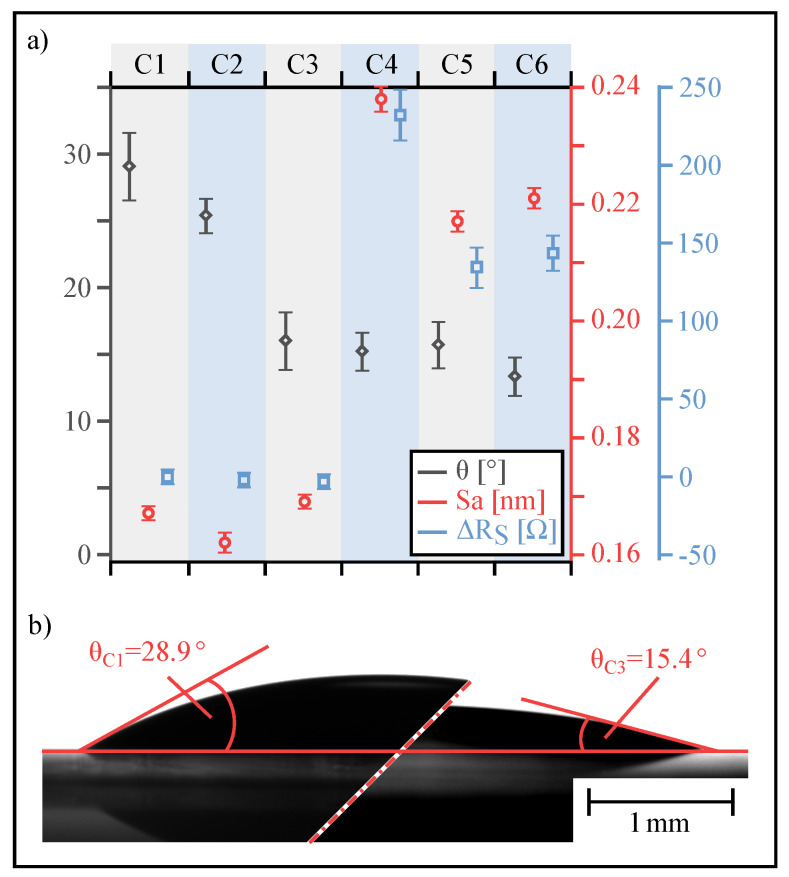
(**a**) Experimental characteristics of six differently treated substrates. Grey rhombs = contact angles, red circles = surface roughness and blue squares = difference in sheet resistance relative to untreated substrate. Each column represents one specific treatment. Left to right: column 1 (C1) untreated, C2 baked for 10 min, C3 baked for 10 min and coated with TiPrime, C4 RIE with O_2_ (100 %, 5 min, 30 W), C5 RIE with O_2_/CF_4_ (25 % O_2_/75 % CF_4_, 5 min, 30 W), C6 analogue C5 and coated with TiPrime. (**b**) Picture of contact angle measurement. Left, contact angle of an untreated substrate (C1). Right, contact angle of a substrate baked for 10 min. and coated with TiPrime (C3).

Figure 5 summarizes the obtained values for the contact angle θ (grey), surface roughness S_a_ (red), and sheet resistance R_s_ (blue) for six differently treated substrates C1 to C6, in which C1 represents the untreated substrate.

The contact angle measurements are performed with an optical contact angle measurement setup OCA 25 (DataPhysics Instruments GmbH, Filderstadt, Germany), the surface roughness is measured with an atomic force microscope (AFM) Dimension Icon (Bruker Nano Surfaces Division, Karlsruhe, Germany) and the sheet resistance is determined with a multimeter 34410A (Agilent Technologies Germany GmbH, Waldbronn, Germany) equipped with a substrate holder, built in-house, in order to obtain comparable measurement results. Each coulumn depicts the measured parameter set for a specific substrate treatment.

Apparently, any of the applied surface treatments (C2–C6) reduces the contact angle, which is determined to be around 30${}^{\circ }$ for the untreated surface (C1). While simply baking the primary substrate results in only a slight decrease of the contact angle (C2), all other surface treatments significantly reduce θ. In particular, baking, including coating with TiPrime (C3), reactive ion etching using O_2_ or O_2_/ CF_4_ (C4 & C5), or a combination of baking and RIE (C6) clearly reduce the contact angle by up to 50 % and, therefore, considerably improve the wetting behavior of the photoresist, advancing the aspired process.

Regarding the surface roughness of the different substrates, simply baking (C2) or baking and coating with TiPrime (C3) shows no distinct effects, as the resulting surface remains rather smooth, i.e., the impact on the surface structure is rather weak and the ITO layer remains intact. In contrast, reactive ion etching steps (C4–C6) lead to an significant increase of the surface roughness from, e.g., S_a_ = 0.162 nm for C2 up to 0.238 nm for C4, a trend being in accordance to [36]. This increase in S_a_ is associated with destructive processes within the ITO layer.

For further processing (electroplating), not only the wetting behavior of the photoresist is of high importance, but also the electrochemical behavior of the ITO electrode. Analogue to the application of ITO electrodes in photovoltaic cells, low sheet resistances result in an increase of the electrochemical device efficiency [37,38]. As chemical treatments of ITO electrodes influence the electrochemical behavior [36,39], differences in sheet resistances can be seen. Again, simply baking or coating the substrate (C2 and C3) yields no considerable change in sheet resistance (cf. Figure 5 right blue axis and ΔR_s_ results). In contrast, surface treatment with RIE leads to a clear resistance increase. According to Yu et al. [40], oxygen plasma treatment (C4) leads to the formation of local surface dipoles; therefore, the working function, the electrochemical state, and, consequently, the electrode properties are affected. The use of O_2_/CF_4_ plasma also increases the sheet resistance. This is due to the fact that this kind of plasma treatment allows for creation of ion damage and incorporation of CF_x_ into the ITO surface. Therefore, a degradation of electrode performance is also detected [41].

As an interim summary on surface treatment, baking and coating with TiPrime (C3) leads to the most suitable results for further processing. Most importantly, the surface remains intact (low S_a_), the wetting behavior improves (θ decreases), and the electrochemical properties for the following electrochemical Au-deposition remain undisturbed (ΔR_s_ remains unaffected on a low level). Furthermore, the process steps are rather simple (baking and coating).

### 4.2. Sticking and Inhibition Layer

**Figure 6:**
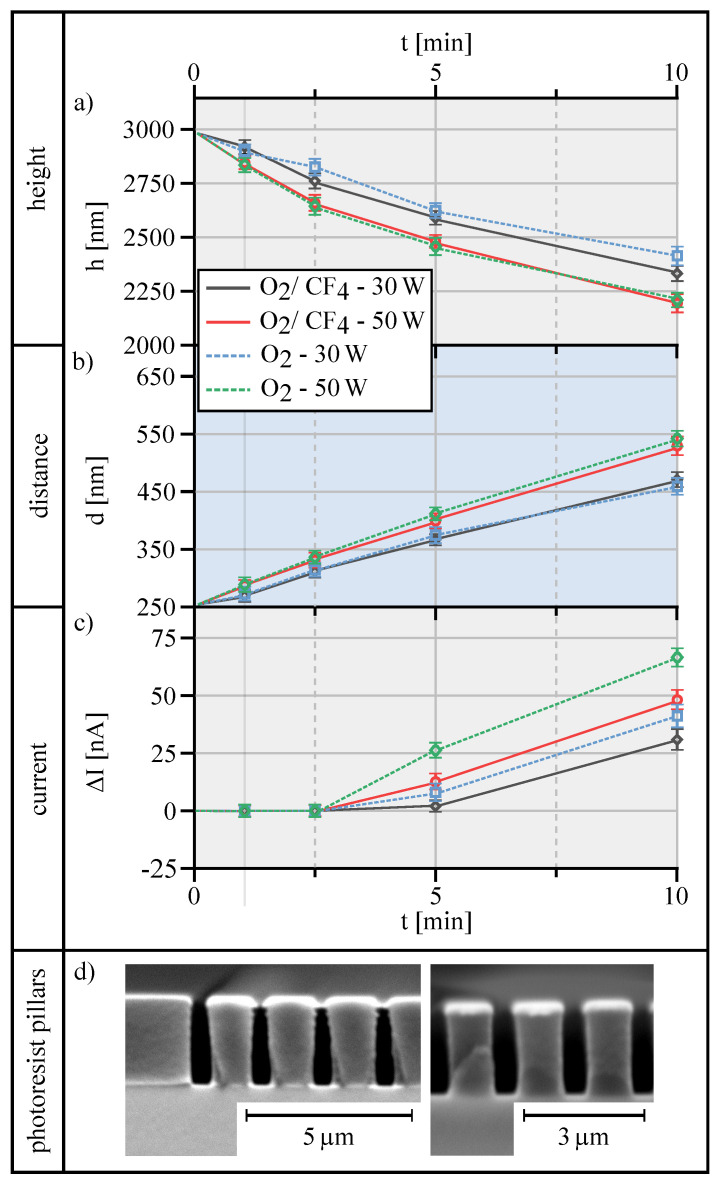
(**a**–**c**) effect of different etching parameters: process gas O_2_ shown as dotted lines, mixed process gas 25 % O_2_/ 75 % CF_4_ in solid lines. 30 W power corresponds to the black line (mixed gas) and the blue dotted line (pure O_2_). 50 W corresponds to the red solid line (mixed gas) and the green dotted line (pure O_2_), repectively. In (**a**) the pillar height versus etching time is depicted, whereas (**b**) shows the correlation between etching time and resulting distance in between the pillar structure. The effect of etching on the electrochemical properties of the ITO layer is shown in (**c**) (for details see Section 4.2). Bottom: (**d**) REM image of the untreated pillar structure (left) and the final RIE treated structure (right).

As the next steps in the process chain, the substrates are coated with photoresist, exposed by 3D DLW, and developed. However, after developing the photoresist structures, two different effects occur: (i) the photoresist pillars tend to stick together at the top (see Figure 6d left) and (ii) in between the pillars an inhibition layer is formed at the ground, which electrically shields the underlying ITO layer.

The sticking of the freestanding photoresist pillars at the top prevents the area between the pillars, denoted here as channels, from being filled with the electrolyte and, in addition, reduces the continuous exchange of used and unused electrolyte. Furthermore, the inhibition layer prevents electrical contact between the electrolyte and the ITO electrode and, thus, counteracts the electrochemical layer deposition. By the use of reactive ion etching, the channels on top are broadened, thus facilitating the electrolyte solution in order to flow into the channels during the electrochemical treatment. In addition, the basic inhibition layer is removed, which prevents contact between electrolyte and ITO electrode.

Figure 6 summarizes and illustrates several effects of four different RIE treatments. Used process gases were 100 % O_2_ (dotted lines) and a mixture of 25 % O_2_ and 75 % CF_4_ (lines), applied for two process powers of 30 W and 50 W (red and green), respectively. First, Figure 6a depicts the height of the photoresist pillars versus the duration of the RIE process, revealing a continuous decrease for all RIE steps starting from 2970 nm for the original pillar after development of the resist. As expected, for both process gases, the height decreases with the applied RIE power, namely from about 20 % in ten minutes for 30 W to 25 % for 50 W.

Figure 6b shows the distance between the individual pillars as a function of RIE process duration for the same RIE process conditions contemplated before in Figure 6a. Because these channels will be filled with gold during the following electroplating step, this distance commensurates with the later gold lattice dimension. Because the thickness of the gold structure, in turn, directly affects the optical transmission properties, this distance should be rather small. The obtained results reveal that the distance between individual pillars increases almost linearly with the duration of the etching process, regardless of the employed process gas, yet with a higher slope for higher RIE power. Specifically, for a 30 W RIE treatment (blue and black) the distance between individual pillars widens from 250 nm to 450 nm, whereas a 50 W treatment results in a pillar distance of 550 nm. As an appropriate compromise, an etching time of 5 min at 30 W leads to a good geometric basic structure of pillars with 2632 nm high and a distance of 367 nm.

On the other hand, the etching process must continue as long as to ensure the complete removal of the inhibition layer in between the pillar structures. Awkwardly, after the complete removal of the inhibition layer, etching will strongly affect the underlying ITO layer and probably destroy it (see Section 4.1). However, AFM measurements for evaluating the quality of the ITO layer are performed, as an intact ITO electrode is essential for the following electrochemical gold deposition. Therefore, after RIE treatment, all photoresist pillar structures are removed by using TechniStrip 1316, a common stripper to completely dissolve the photoresist and remove it from the substrate. Consequently, the ITO layer, which was originally underneath the freestanding pillars, should be completely intact. In contrast, areas that are located in the channels in between the original pillar structures should show some effects that result from the preceding etching treatments. However, high precision AFM height measurements reveal no differences in height of the ITO layer at any place, whether the ITO layer was located underneath a pillar structure or in between them. This indicates that no deep physical damage was done by the RIE treatment.

Different current flows throughout the ITO layer can be measured by using a conductive AFM tip and applying a voltage during the measurement. As long as the ITO layer is intact, the resulting current should be the same at any position on the structure. Yet, any damage of the ITO layer should negatively affect the measurable current, which will finally lead to a poorer quality of the electrodeposited gold layer. In order to evaluate the quality of the ITO layer in this respect, the current underneath the pillar structure, as described above dissolved by the stripper, as well as in the channels between the original pillar structure, i.e., areas that were expose to the RIE process, are measured. The difference between these currents ΔI is regarded as a qualitative measure of the integrity and quality of the ITO layer with respect to the forthcoming electroplating process step and it is plotted in Figure 6c as a function of the (RIE) etching time.

The results for ΔI in Figure 6c show no differences up to 2.5 min. etching time (ΔI = 0), i.e., an intact ITO layer can be assumed at every location on the surface. After 5 min. of etching, however, ΔI increases, whic indicates a degradation of the ITO layer and its electrical properties between the original pillar structures. The longer the etching endures, the higher this damage to the ITO layer, which implies poor electrochemical performance during the intended next process step. The results that are shown in Figure 6c also imply that this effect gets stronger with increasing RIE power, and it is more pronounced for O_2_ as compared to O_2_/CF_4_ process gases. Altogether, it appears that etching up to 5 min. using 30 W and O_2_/CF_4_ is a proper compromise to remove the inhibition layer while assuring low abrasion of the ITO layer and, thus, maintaining its electrical function for the electrochemical deposition.

Finally, Figure 6d depicts the SEM images of the photoresist pillars directly after development (left) and after RIE treatment (right). Apparently, directly after developing the freestanding structures almost stick together at the top (left). In addition, the formation of the inhibition layer at the interface with the substrate is also clearly visible. In comparison, the right image clearly shows that the distances between the photoresist pillars are widened and the inhibition layer is removed.

These comprehensive results on the RIE treatment in order to remove the inhibition layer and to eliminate the sticking of adjacent pillars are summarized as follows. The height of the pillars can be well definable reduced by controlling the etching duration in conjunction with the applied power. The particular kind process gas (O_2_ or O_2_/CF_4_) has only minor influence (Figure 6a). The same holds for the widening of the channels between pillar rows (Figure 6b), yet, under any condition, the height reduction is more pronounced as the widening of the channels, because the widening can only start after the shielding pillar tops are removed (cf. Figure 6d, left). Nonetheless, 5 min. etching using 30 W appears to sufficiently open access to the channels in between the pillars with acceptable broadening of the channel width. According to Figure 6c, these conditions also comply with the necessity of removing the inhibition layer without damaging the ITO, when employing 25 % O_2_/75 % CF_4_ process gas.

### 4.3. Weak Adhesion (Part II)

**Figure 7:**
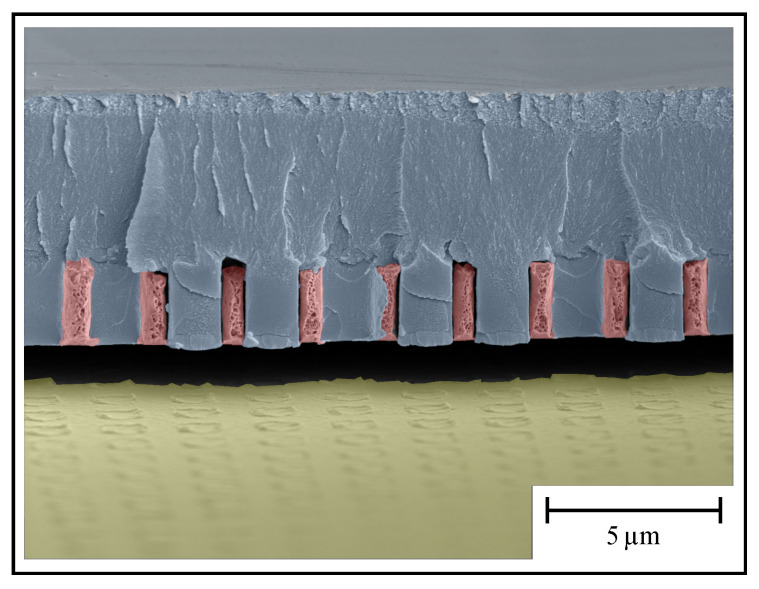
Colorized SEM image of the fixation, side view at an angle of 35${}^{\circ }$. The red colored areas show the metallic structure, the blue area shows both the freestanding photoresist pillars as well as the fixation layer, and the yellow area shows the substrate. Please note, during preparation for taking this SEM image (by cleaving to allow a side view) the metallic HWA structure as well as the photoresist is detachted from the substrate.

After optimization of the photoresist adhesion (Section 4.1) and after successful preparation for the galvanic gold deposition, the pillars are removed by the second developing step depicted in Figure 3. Unfortunately, the deposited gold layer is often detached during this step (Figure 4, bottom left). In order to stabilize the gold layered structure, before removing the pillars, a second photoresist layer is applied on the complete surface of the sample. This layer is exposed to UV-light irradiation at a circular area of 2 mm in diameter. After the consecutive developing step, in this circular area, the fixation layer, as well as the photoresist pillar structures, are removed. The outer residual fixation layer acts as a supportive film, so the bare gold structure in the center can be optically characterized. Figure 7 shows the fixation of the metallic hollow waveguide array (HWA) structure on top of the substrate.

## 5. Optical Experiments

The waveguide dimensions (w_x_ & w_y_) and the width of the metal side walls (d_x_ & d_y_) are measured while using a MAIA3 scanning electron microscope (Tescan GmbH, Dortmund, Germany). In order to determine the waveguide geometry, a total of 25 waveguides are measured at random positions over the entire HWA. For the quarter-wave plate, the dimensions of the hollow waveguides are w_x_ = 1403 ± 7 nm and w_x_ = 815 ± 3 nm. The half-wave plate has the same dimension w_x_ = 1403 ± 7 nm in the x-direction as the quarter-wave plate. Only in the y-direction w_y_ = 883 ± 4 nm both polarization converters differ. For both polarization converters, the dimensions of the metal sidewalls are d_x_ = 396 ± 6 nm and d_y_ = 421 ± 4 nm. The waveguide geometries from the fabricated HWA show excellent agreement with the targeted values of w_x_ = 1400 nm and w_y_ = 814 nm (quarter-wave plate) as well as w_x_ = 1400 nm and w_y_ = 884 nm (half-wave plate). However, the width of the metal sidewalls does not influence the polarization behavior of the polarization converters, only the transmission depends on the width of the sidewalls. While the transmission of the quarter-wave plate is 47.8 ± 0.13 %, the transmission of the half-wave plate amounts to 51.3 ± 0.18 %. These can be assigned by the different waveguide dimensions of w_y,λ/4_< w_y,λ/2_. The proportionate higher amount of metal sidewalls on the same square area reduces the transmission due to the smaller waveguide width w_y_ of the quarter-wave plate.

**Figure 8:**
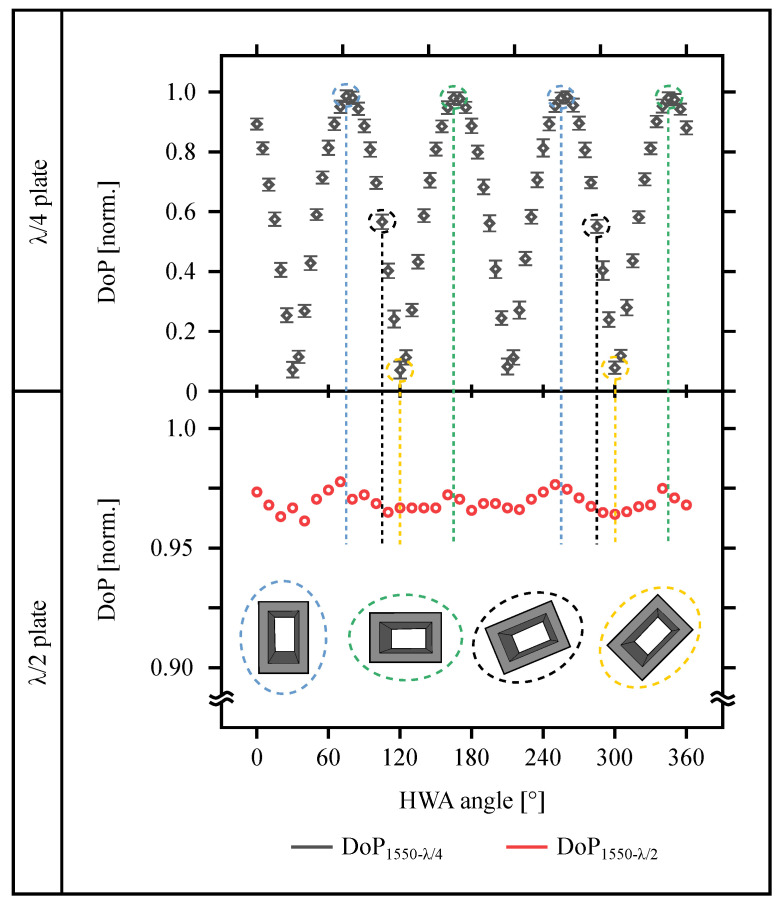
Degree of Polarization against the hollow waveguide array (HWA) angle for a quarter-wave plate (top) and a half-wave plate (bottom). For a clear presentation, the results for the half-wave plate is only plotted in steps of 10${}^{\circ }$. The schematic drawings of the hollow waveguides in the figure show the orientation of the waveguides for selected positions. These positions are marked in color in the figure. Please note, because of the smaller amount of measurement values for the half-wave plate, no measured value is given for some positions.

The characterization of the fabricated hollow waveguide arrays is done by measuring the minimum and maximum transmitted power and calculate the degree of polarization. The measurement setup consists of a linear polarized laser diode emitting at a wavelength of λ = 1550 nm in constant mode. A beam condensor system is used in order to reduce the beam diameter to 1 mm and collimate the beam. The hollow waveguide array is mounted in a rotatable holder. After passing through the HWA, the minimum and maximum power is set with an analyzerthat is mounted in a rotatable holder and measured with a power meter. The angle of the hollow waveguide array is stepwise changed from 0${}^{\circ }$ to 360${}^{\circ }$ in steps of 5${}^{\circ }$. For every step, the degree of polarization is calculated by

(4) $$DoP=\frac{P_{max}-P_{min}}{P_{max}+P_{min}}$$

and the results for both the quarter- and the half-wave plate are shown in Figure 8.

If the degree of polarization assumes a value of 1, the field after the hollow waveguide array is completely linear polarized. The power transmitted through the analyzer is maximum in the transmission direction and, for a crossed analyzer, the beam is almost completely blocked. The difference between ($P_{max}$) and ($P_{min}$) is maximal and the degree of polarization is calculated according to Equation (4) to 1. For a value of 0, the field after the HWA is circularly polarized. A circular polarization has no preferred direction and the transmitted power is equal for every position of the analyzer. According to Equation (4), the degree of polarization is calculated as 0. For each value between 0 and 1, the field after the HWA is elliptically polarized.

The measurements presented in Figure 8 for the quarter-wave plate show four maxima and four minima. The maxima occur when the hollow waveguide array is aligned vertically or horizontally (the blue and green circled hollow waveguides shown in Figure 8, bottom). In the hollow waveguide, only one mode is excited by the linearly polarized input field, no phase delay occur and the linearly polarized input field remains linearly polarized after the hollow waveguide array. The minima occur when the hollow waveguide array is aligned at an angle of 45${}^{\circ }$ with respect to the horizontal or vertical (the yellow circled hollow waveguide shows two of these four positions). For these four positions, the two modes in the hollow waveguide are equally excited and the phase delay is maximal. The maximum phase delay for a quarter-wave plate is 90${}^{\circ }$, or like shown in this work, 270${}^{\circ }$. Relating to these values, the linear polarized input field becomes to a circular polarized output field. Every other position of the hollow waveguide array between the positions mentioned above, the field is elliptically polarized. Both of the waveguide modes (horizontal and vertical) are excited with different strengths and depending on the angular position of the HWA, the ellipticity of the polarization state changes. For the half-wave plate (Figure 8, bottom), the degree of polarization remains close to 1 and no distinct extreme values are observable. The maximum phase delay for a half-wave plate is 180${}^{\circ }$. The linear polarized input field persist linear polarized after the hollow waveguide array, but the field changes their orientation (e.g., a horizontal linear polarized input field becomes a vertical linear output field).

## 6. Conclusions

We have presented a femtosecond three-dimensional direct laser writing based subractive-additive hybrid fabrication process of metallic structures with lateral dimensions of less than 400 nm and they have exemplified the capabilities of this technological approach by realizing and demonstrating the performance of a fully functional hollow waveguide array based polarization element. In order to facilitate high precision and high quality of such micro-nanostructured components, we comprehensively studied the technological challenges of such sophisticated fabrication process and identified solutions to these. In particular, an appropriate pretreatment of the substrate significantly improves the adhesion of non-metallic freestanding photorosist pillars with rectangular dimensions of 814 nm × 1400 nm for the quarter-wave plate, 884 nm × 1400 nm for the half-wave plate, both with a height of 3000 nm, respectively. After developing, a thin inhibition layer forms on the ITO surface and prevents contact between the ITO electrode and the electrolyte, hampering electrochemical deposition. Additionally, the freestanding photoresist pillars tend to stick together at the top because of their high aspect ratio. Both of the challenges are solved with a carefully adjusted reactive ion etching step, removing the occurring inhibition layer and increasing the gap between the pillars. Subsequently, the additive manufactured metallic structure is fixed with a second photoresist layer on top of the substrate. Furthermore, an optical characterization of the two wave plates is performed over their complete angular range and the functionality of a quarter- and half-wave plate that is based on hollow waveguides is shown.
